# Supplemental Upward Lighting from Underneath to Obtain Higher Marketable Lettuce (*Lactuca sativa*) Leaf Fresh Weight by Retarding Senescence of Outer Leaves

**DOI:** 10.3389/fpls.2015.01110

**Published:** 2015-12-14

**Authors:** Geng Zhang, Shanqi Shen, Michiko Takagaki, Toyoki Kozai, Wataru Yamori

**Affiliations:** ^1^Graduate School of Horticulture, Chiba UniversityMatsudo, Japan; ^2^Center for Environment, Health and Field Sciences, Chiba UniversityKashiwa, Japan; ^3^Japan Plant Factory AssociationKashiwa, Japan; ^4^Precursory Research for Embryonic Science and Technology, Japan Science and Technology AgencyKawaguchi, Japan

**Keywords:** LED, light color, supplemental upward lighting, photosynthesis, plant factory, supplemental lighting

## Abstract

Recently, the so-called “plant factory with artificial lighting” (PFAL) approach has been developed to provide safe and steady food production. Although PFALs can produce high-yielding and high-quality plants, the high plant density in these systems accelerates leaf senescence in the bottom (or outer) leaves owing to shading by the upper (or inner) leaves and by neighboring plants. This decreases yield and increases labor costs for trimming. Thus, the establishment of cultivation methods to retard senescence of outer leaves is an important research goal to improve PFAL yield and profitability. In the present study, we developed an LED lighting apparatus that would optimize light conditions for PFAL cultivation of a leafy vegetable. Lettuce (*Lactuca sativa* L.) was hydroponically grown under white, red, or blue LEDs, with light provided from above (downward), with or without supplemental upward lighting from underneath the plant. White LEDs proved more appropriate for lettuce growth than red or blue LEDs, and the supplemental lighting retarded the senescence of outer leaves and decreased waste (i.e., dead or low-quality senescent leaves), leading to an improvement of the marketable leaf fresh weight.

## Introduction

Population growth has led to a steady increase in the demand for food, and now poses a threat to food security. In recent years, air pollution, rapid population growth, and resource shortages have focused increasing attention on food security. An emerging industry with the potential to alleviate some of these problems takes advantage of what have been called “plant factories”, which can produce high-yield and high-quality plants using less water, nutrients, land, and labor than is possible with conventional agriculture (Kozai, 2013a; Hu et al., 2014; Yamori et al., 2014). Plant factories with artificial lighting (PFALs) create an enclosed cultivation system that allows control of environmental factors such as lighting, temperature, humidity, and CO_2_ concentration. Moreover, PFALs can overcome adverse conditions such as heavy rain, heavy snow, strong winds, and temperature extremes. PFALs have been already commercially used for the production of leafy vegetables in Japan, China, and Taiwan (Kozai, 2013b).

Light sources such as fluorescent lamps, metal-halide lamps, and high-pressure sodium lamps are generally used for plant cultivation. They are used to increase the photosynthetic photon flux density (PPFD), but they also provide wavelengths that are not used efficiently or at all to support photosynthesis and plant growth (McCree, 1972; Björkman, 1981). In comparison, LED lighting systems have several advantages, including greater wavelength specificity (i.e., a narrow bandwidth), long operating lifetimes, and less heating. However, the optimal light wavelengths for plant cultivation remain unclear. Numerous studies have suggested that red and blue light are the most useful wavelength bands to drive photosynthesis, since chlorophyll has its maximum absorption in those bands (e.g., McCree, 1972; Okamoto et al., 1996), but blue light also plays an important photomorphogenic role (e.g., suppresses hypocotyl elongation; Goins et al., 1997; Massa et al., 2008) in plants. However, it has been shown that the optimal light color for plant growth differs among plant species (Kim et al., 2006). For example, lettuce (*Lactuca sativa* L.) grown under red LEDs developed more leaves than lettuce grown under blue LEDs (Yanagi et al., 1996), but for spinach (*Spinacia oleracea* L.) or radish (*Raphanus sativus* L.) growth, the use of only red LEDs was unsuitable (Yorio et al., 2001). Other studies have found that light sources that contain blue light improved dry matter production and the photosynthetic capacity in pepper (*Capsicum annuum* L.; Brown et al., 1995), wheat (*Triticum aestivum* L.; Goins et al., 1997), and spinach (*Spinacia oleracea* L.; Matsuda et al., 2007). Thus, it remains unclear what light source would be most suitable for plant cultivation in a PFAL system. It is therefore necessary to identify the optimal light sources for various species to maximize plant yields.

On the other hand, although PFALs can produce high yields, the high plant density creates suboptimal conditions, because the outer or lower leaves are shaded and therefore senesce faster. Owing to shading by upper or outer leaves and by neighboring plants, leaves beneath the plant canopy suffer from low light conditions (Terashima et al., 2005). The leaf senescence that occurs at low light intensity is accompanied by chlorophyll loss, degradation of photosynthetic proteins, a decline in photosynthetic activity, and the remobilization of nutrients to younger tissues (Gan and Amasino, 1997; Weaver and Amasino, 2001; Brouwer et al., 2012). The senescent leaves become visibly yellow (chlorotic) and wilted (McCabe et al., 2001), leading to a reduction of the market price. Thus, these leaves must be removed, which can significantly decrease plant yield and increase labor costs. Therefore, establishing cultivation methods that retard senescence of outer leaves is an important goal to improve yield and profitability.

Since the main problem is the low light conditions experienced by shaded leaves, improving the light conditions of these leaves could delay senescence. Previous studies have shown that irradiation of both the adaxial and abaxial sides of a leaf can increase photosynthesis (Terashima, 1986; Soares et al., 2008), and different light colors have different effects on leaf senescence (Causin et al., 2006). However, no studies have examined the effects of supplemental upward lighting of the abaxial sides of leaves from underneath the plant to delay senescence of shaded leaves and improve plant growth. The present study therefore had two purposes: to study the effect of light color on photosynthesis and plant growth of romaine lettuce, and to analyze the effect of supplemental upward lighting from underneath the plant on leaf senescence in the outer leaves. We also analyzed the economic benefits of this technique. We found that white LEDs were more appropriate for romaine lettuce than red or blue LEDs, and that the supplemental upward lighting retarded senescence of the outer leaves and reduced waste, leading to an improvement in marketable leaf fresh weight; it also improved the nutrient quality of the plants.

## Materials and Methods

### Plant Materials and Growth Conditions

**FIGURE 1:**
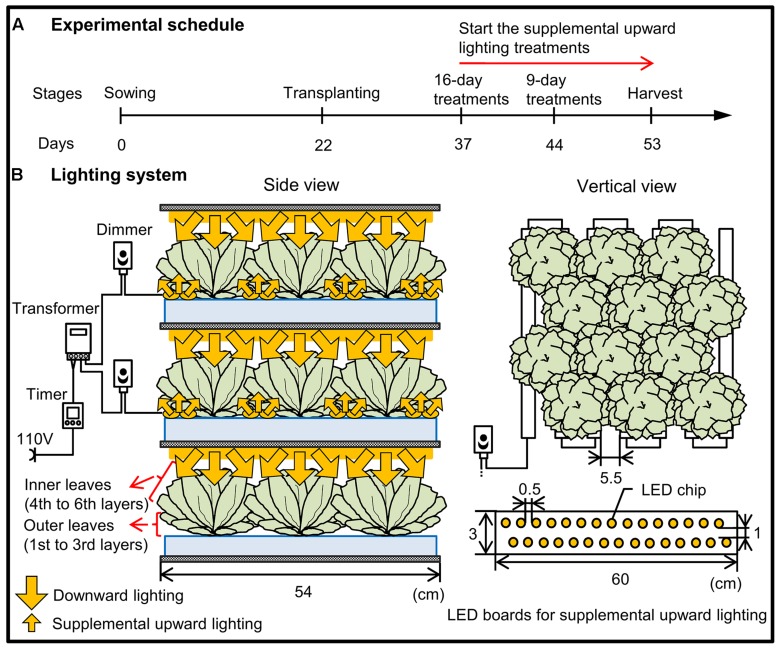
**Schematic diagram of the experimental schedule **(A)** and the lighting system **(B)** used in the present study.** **(A)** At 22 days after sowing, the seedlings were transplanted into growth chambers equipped with 200 μmol m^-2^ s^-1^ PPFD of downward-facing white, red, or blue LEDs and with light provided for 16 h per day. The supplemental upward lighting treatments were performed from 15 or 22 days after transplanting until harvest. **(B)** There were three vertically arranged cultivation beds for each light color. Lettuce grown in the top and middle layers received light from above (downward lighting), with supplemental upward lighting for 9 and 16 days, respectively. Lettuce grown in the bottom layer received light only from above, without any supplemental upward lighting. The treatment arrangement was the same for each LED color: lettuce was grown at 37 plants m^-2^, with 22 LED boards (each board containing two rows of LED chips) m^-2^ used for supplemental upward lighting. A digital timer, dimmer, and transformer were used to maintain the light period (16 h, the same as the downward lighting) and light intensity (40 μmol m^-2^ s^-1^ PPFD).

The experiment was conducted in a commercial plant factory, which has two cultivating compartments: one is a nursery room and the other is a cultivation room. Romaine lettuce (*Lactuca sativa* L. var. Romana; Takii Seed Co., Kyoto, Japan) seeds were sown in sponge blocks (W 2.3 cm × D 2.3 cm × H 2.7 cm), and the seedlings were grown in the nursery room at 20/17°C (day/night) under a PPFD of 350 ± 10 μmol m^-2^ s^-1^ for 12 h provided by cool white fluorescent lamps. At 22 days after sowing, the seedlings were transplanted into the cultivation room equipped with 200 μmol m^-2^ s^-1^ PPFD of downward-facing white LEDs, red LEDs (peak wavelength 660 nm), or blue LEDs (peak wavelength 450 nm; **Supplementary Figure S1A**; red and blue LEDs were provided by Shibasaki Inc., Saitama, Japan; white LEDs were provided by ODC Co., Ltd., Kanagawa, Japan). Plants were grown in a deep-flow hydroponic system (37 plants m^-2^) in Enshi formula nutrient solution with an electrical conductivity of 1.7 ± 0.1 dS m^-1^ and a pH of 6.8 ± 0.2. The air temperature was maintained at 25/20°C (day/night), the relative humidity at 60%, the photoperiod at 16 h, and the CO_2_ concentration at 1000 ppm. We chose 1000 ppm CO_2_ because this is the most common concentration used in Japanese PFALs (Kozai, 2013a). The supplemental upward lighting treatments were performed from 15 days (when all outer leaves became shaded) or 22 days (when visible senescence of outer leaves began) after transplanting, with illumination at 40 μmol m^-2^ s^-1^ PPFD at the height of the outer leaves (4.0 ± 0.5 cm). The illumination was provided by supplemental LEDs placed on cultivation panels in order to direct the light upward from underneath the plants (**Figure 1**). The light colors of these supplemental LEDs were same to those of the LEDs used for downward lighting from above (**Supplementary Figure S1B**).

### Measurements of Gas Exchange and Chlorophyll Fluorescence

Gas exchange was measured with a portable gas exchange system (LI-6400; Li-Cor Inc., Lincoln, NE, USA) as described previously (Yamori et al., 2009, 2010). Plants were divided into six layers of leaves. Counting from the lowest leaf, the first to third layers of leaves (white light, 10–11 leaves per plant; red light, 10–11 leaves per plant; blue light, 5–6 leaves per plant) were the outer leaves, and the fourth to sixth layers of leaves (white light, 9–10 leaves per plant; red light, 8–9 leaves per plant; blue light, 6–7 leaves per plant) were the inner leaves. After 30 min of illumination to obtain steady-state photosynthesis, the net photosynthetic rate of the inner leaves (sixth layer) and outer leaves (third layer) were measured.

To evaluate the degree of leaf senescence, we measured the maximum potential photochemical efficiency (the ratio of variable to maximum fluorescence, *F*_v_/*F*_m_) using an Imaging-PAM fluorometer (Walz, Effeltrich, Germany). Leaf disks (1.3 cm in diameter) were taken from the outer and inner leaves of each treatment (from the first to sixth layers), and were then vacuum-infiltrated in deionized water that included 0.005% Tween 20 (Shao et al., 2013). *F*_v_/*F*_m_ was measured after 30 min of incubation in darkness.

### Determinations of Chlorophyll, Carbon, and Nitrogen Contents

Right after the gas exchange and chlorophyll fluorescence measurements, leaf disks (0.85 cm in diameter) were taken from the outer and inner leaves (same leaves with the measurements of *F*_v_/*F*_m_) of each treatment. Chlorophyll was extracted in *N,N*-dimethylformamide and its content was determined using a spectrophotometer, according to the procedure of Porra et al. (1989). Leaf carbon and nitrogen contents were measured with a Vario EL III elemental analyzer (Elementar, Hanau, Germany) as described by Yamori et al. (2005).

### Plant Growth and Nutritional Quality of the Romaine Lettuce

At 53 days after sowing, plants were harvested, and the leaf angle, leaf number, total leaf area, root length, leaf and root fresh weights, and leaf and root dry weights were measured. Total leaf area was determined using a leaf area meter (LI-300; Li-Cor Inc., Lincoln, NE, USA), and the dry weights of leaf and root were measured after oven-drying at 80°C for more than 72 h.

As a measure of the nutrient quality, the ascorbic acid and nitrate contents in the outer and inner leaves (all leaves in each group of layers were cut into pieces, and the 1 g fresh samples were used for measurements) of plants in each treatment were quantified by using an RQ Flex plus reflectometer (Merck, Darmstadt, Germany), following the method of Pantelidis et al. (2007).

### Electricity Consumption Measurements

The consumption of electrical energy by each LED panel was measured with a multimeter and a clamp ammeter (Hioki 3169-01, Hioki E.E. Corporation, Nagano, Japan), and were used to evaluate the economics of the supplemental lighting.

### Statistical Analysis

Values were compared between illumination treatments by Tukey’s multiple-comparison test (for photosynthetic rates without supplemental lighting, fresh weights, and wastage rates) or Student’s *t*-test (for photosynthetic rates with supplemental lighting and nutrient contents) in SPSS statistical software v. 21.0 (SPSS, Chicago, IL, USA). Differences were considered significant at *P* < 0.05.

## Results

### Leaf Characteristics

**FIGURE 2:**
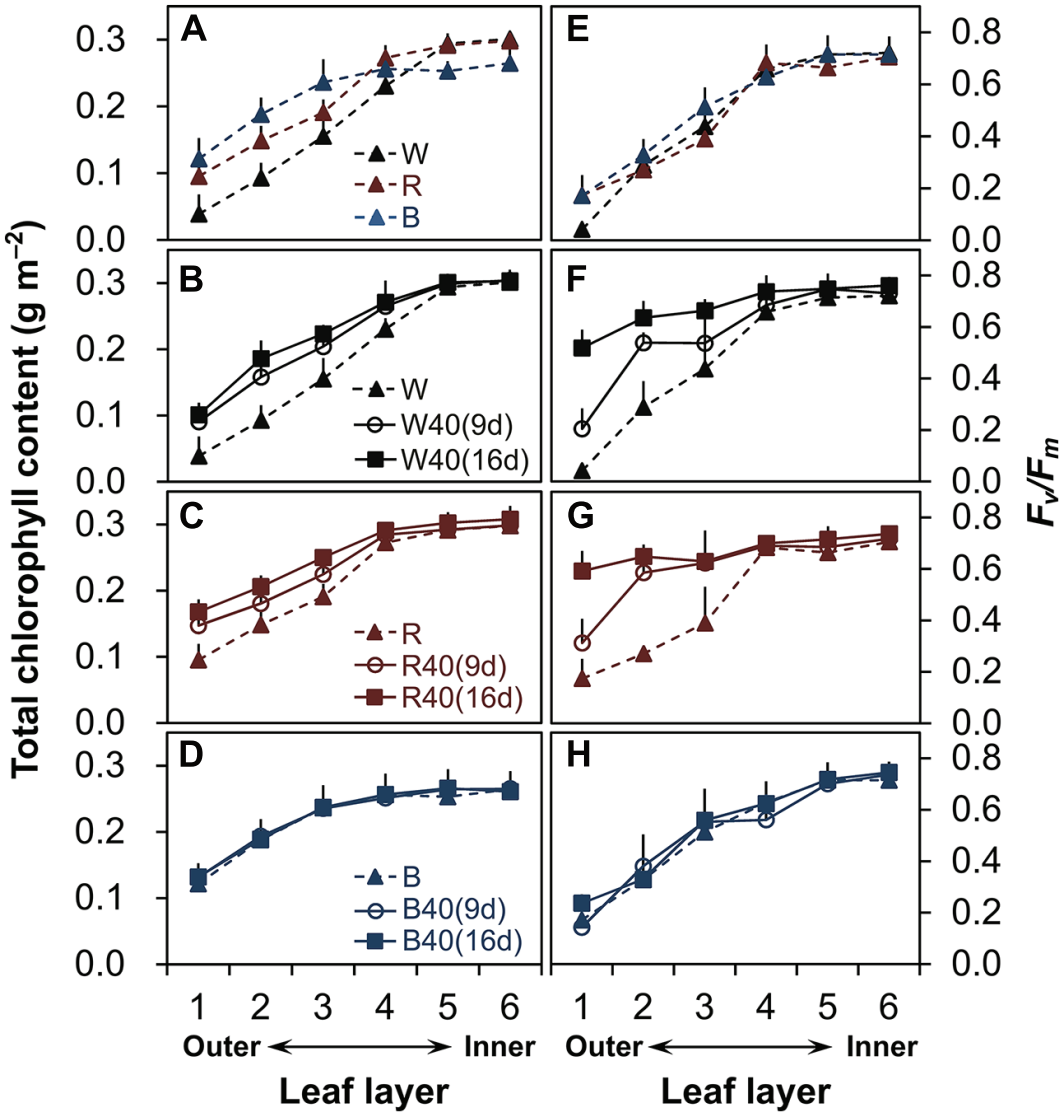
**Total chlorophyll content **(A–D)** and maximum quantum yield (*F*_v_/*F*_m_) **(E–H)** in lettuce leaves from the six layers of leaves in plants grown under downward lighting by white (W), red (R), or blue (B) LEDs, without or with supplemental upward lighting for 9 days (“9d”) or 16 days (“16d”) at 40 μmol m^-2^ s^-1^ PPFD.** Data represent means ± SD (*n* = 3 to 5). **(A,E)** No supplemental upward lighting. **(B–D,F–H)** Same downward lighting as in **(A)**, but with supplemental **(B,F)** white, **(C,G)** red, or **(D,H)** blue upward lighting.

In plants grown under downward lighting but without upward lighting, the total chlorophyll content (**Figure 2A**, **Supplementary Table S1**) and maximum potential photochemical efficiency (*F*_v_/*F*_m_; **Figure 2E**, **Supplementary Table S1**) were highest in the most newly expanded leaves (sixth layer), but gradually decreased from the inner leaves (fourth to sixth layers) to the outer leaves (first to third layers).

However, supplemental upward lighting maintained significantly higher total chlorophyll content and *F*_v_/*F*_m_ values in the outer leaves of plants grown under white LEDs (**Figures 2B,F**, **Supplementary Table S1**) and red LEDs (**Figures 2C,G**, **Supplementary Table S1**), but not under blue LEDs (**Figures 2D,H**, **Supplementary Table S1**), than in plants grown without upward lighting (**Figures 2A,E**, **Supplementary Table S1**). The 9-day supplemental lighting treatments maintained high total chlorophyll content and *F*_v_/*F*_m_ in the outer leaves to some extent, but the 16-day treatments showed a more pronounced effect in retarding senescence of the outer leaves. White or red supplemental lighting also maintained the leaf nitrogen content in the outer leaves (**Supplementary Table S2**). However, blue supplemental lighting (but not red or white) allowed a significant decrease in the leaf nitrogen content in the inner leaves.

### Photosynthesis

**FIGURE 3:**
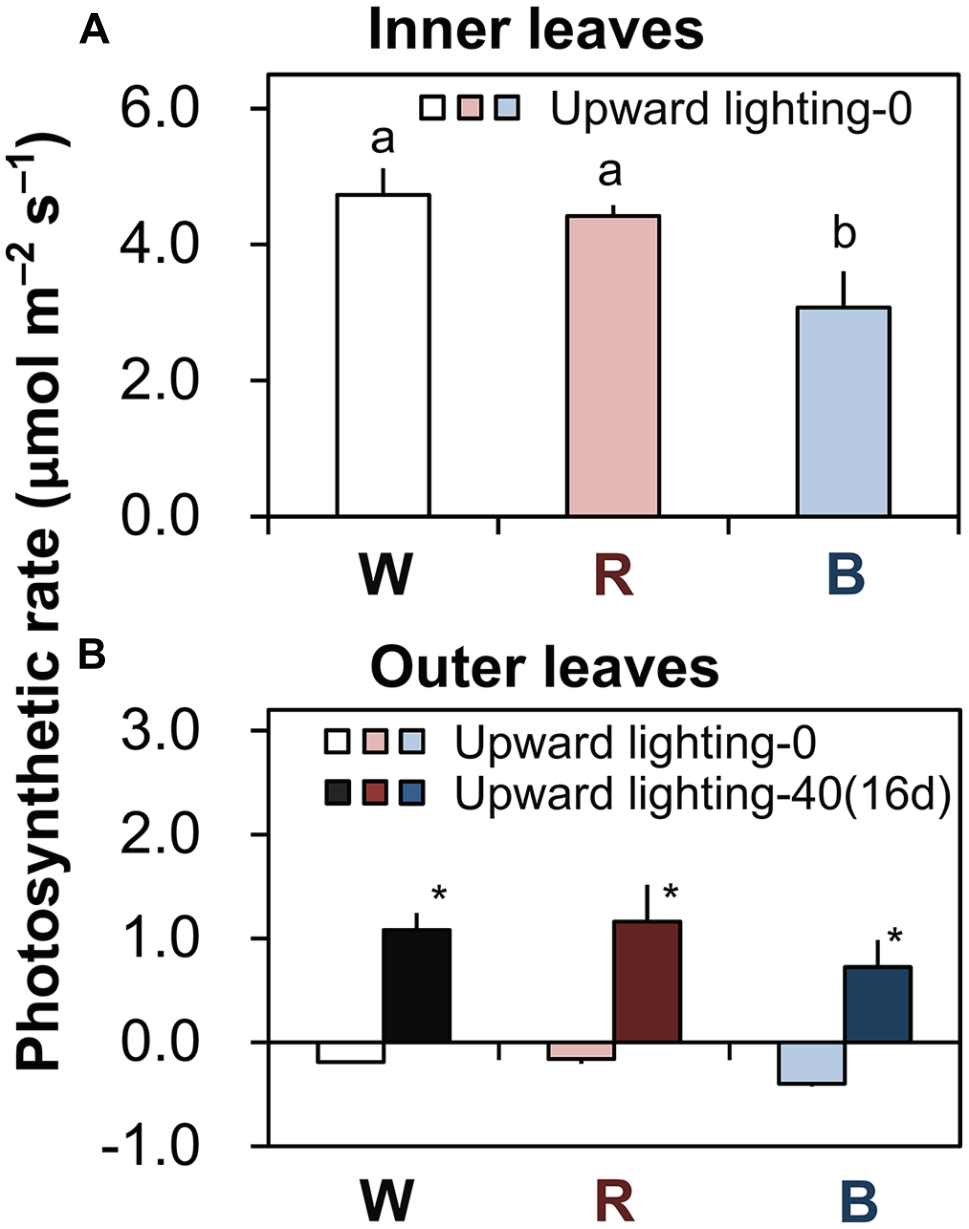
**Photosynthetic rates of plants grown under downward lighting by white, red, or blue LEDs, with or without supplemental upward lighting.** Data represent means ± SD (*n* = 3). **(A)** Photosynthetic rates were measured under ambient light conditions in the most newly expanded leaves (in the sixth layer) in plants grown without upward lighting. Bars labeled with different letters differ significantly (Tukey’s HSD test, *P* < 0.05) among the three light colors. **(B)** Photosynthetic rates were measured under ambient light conditions in the outer leaves (in the third layer) in plants grown under downward lighting by white, red, or blue LEDs, with or without supplemental upward lighting for 16 days. Bars labeled with “^∗^” differ significantly for a given light color (Student’s *t*-test, *P* < 0.05). “Upward lighting-0” denotes plants grown without supplemantal upward lighting; “Upward lighting-40 (16d)” denotes plants grown with 16 days of supplemental upward lighting at 40 μmol m^-2^ s^-1^ PPFD.

In plants grown under downward lighting but without upward lighting, the photosynthetic rates of the most newly expanded leaves (in the sixth layer) were highest in plants grown under white or red LEDs, which did not differ significantly, and were significantly lower under blue LEDs (**Figure 3A**).

With supplemental upward lighting, the outer leaves showed positive net photosynthetic rates. In contrast, without upward lighting, plants had negative net photosynthetic rates in the outer leaves (**Figure 3B**). These results show that supplemental upward lighting could shift the carbon balance from negative to positive (i.e., it improved photosynthesis in the outer leaves).

### Plant Growth

**FIGURE 4:**
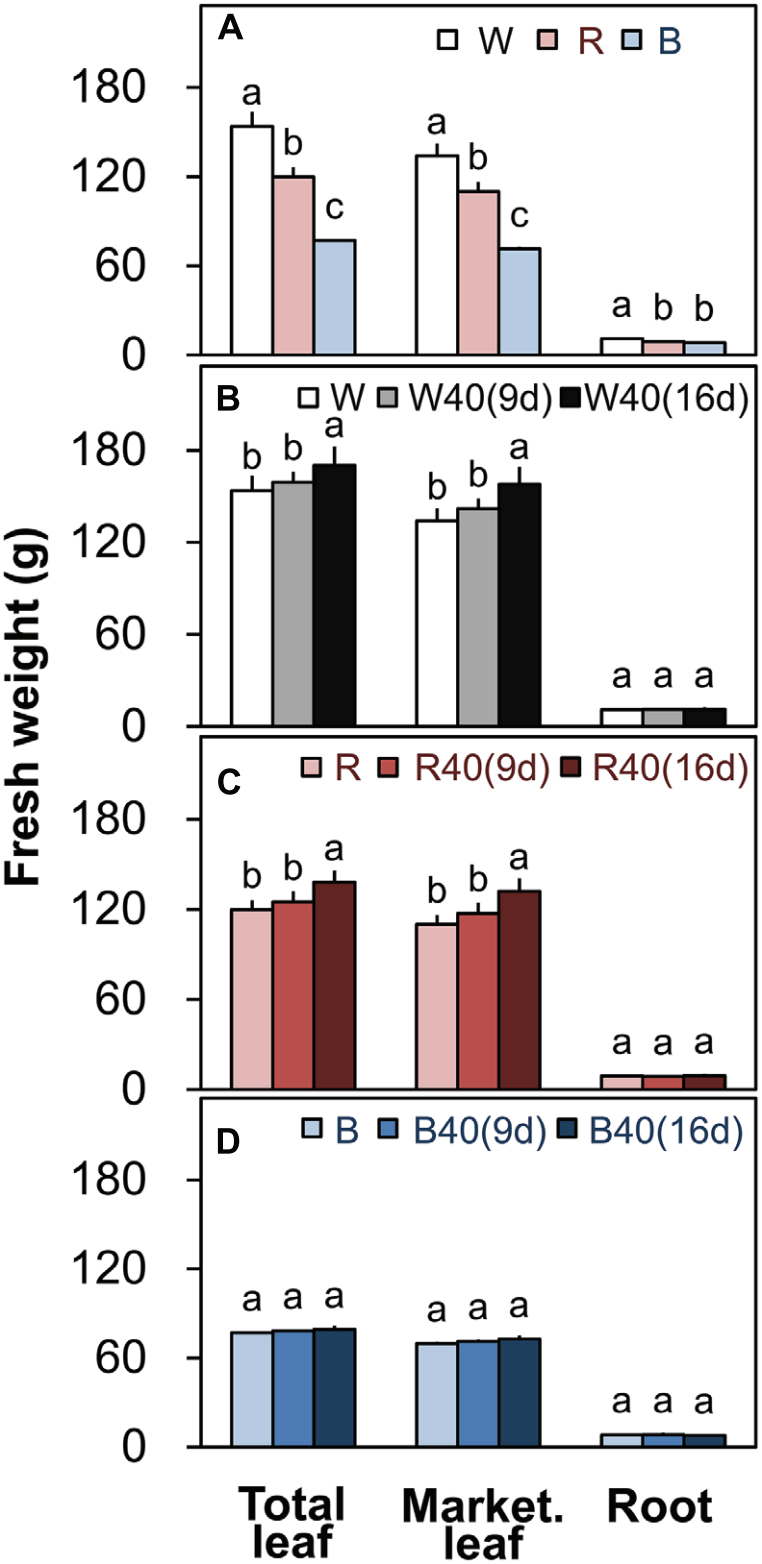
**Total leaf fresh weights, marketable leaf fresh weights, and root fresh weights of plants grown under **(A)** white (W), red (R), and blue (B) LEDs without supplemental upward lighting, or under the same downward lighting from above with supplemental upward lighting from **(B)** white, **(C)** red, or **(D)** blue LEDs.** Data represent means ± SD (*n* = 3 to 5). Supplemental upward lighting was provided for 9 days (“9d”) or 16 days (“16d”) at 40 μmol m^-2^ s^-1^ PPFD. Bars labeled with different letters differ significantly (Tukey’s HSD test, *P* < 0.05).

**FIGURE 5:**
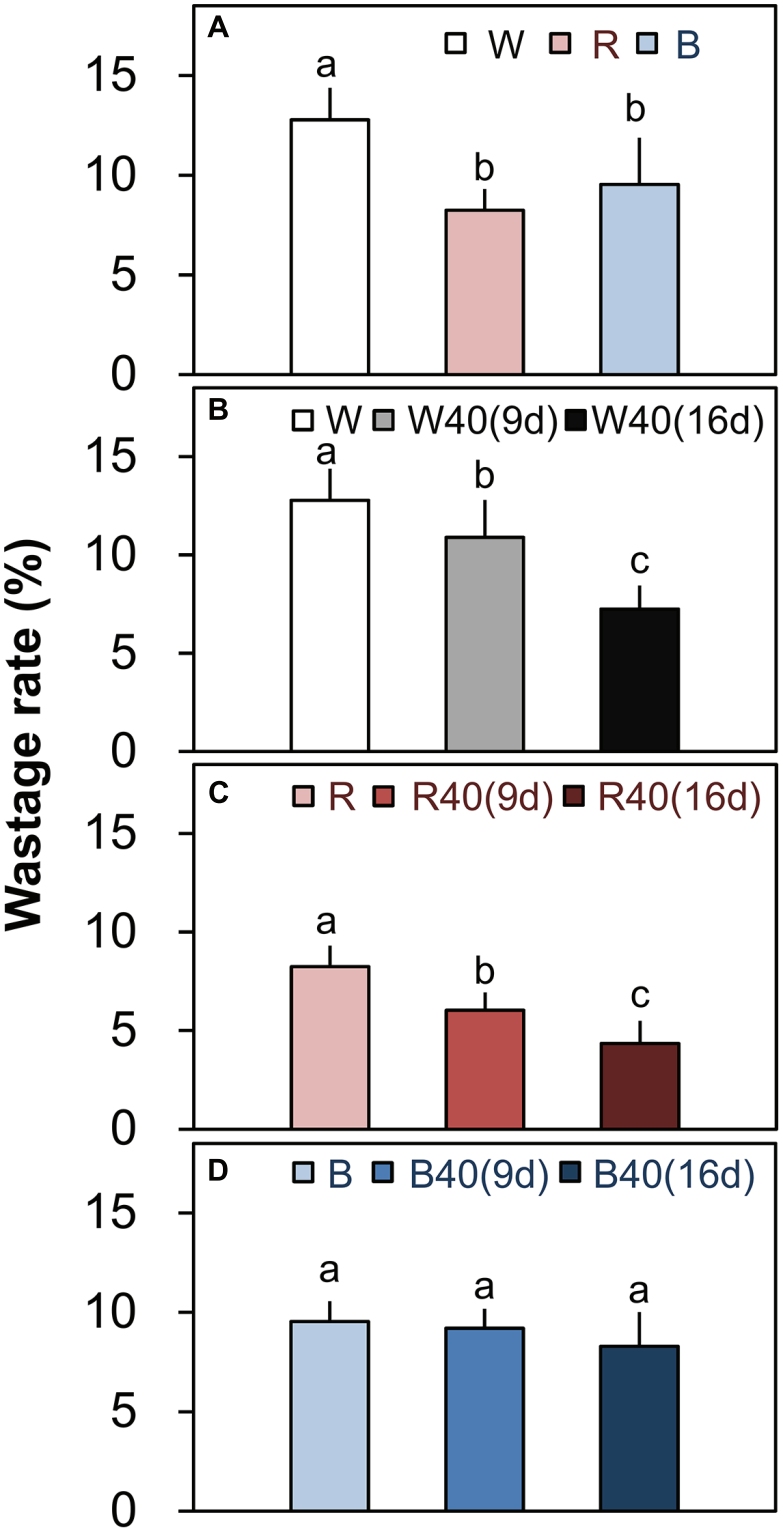
**Wastage rates (total leaf fresh weight minus marketable leaf fresh weight; **Figure 4**) of outer senesced leaves of plants grown under downward lighting by white, red, or blue LEDs **(A)** without and **(B–D)** with supplemental upward lighting.** Data represent means ± SD (*n* = 3 to 5). Supplemental upward lighting was provided for 9 days (“9d”) or 16 days (“16d”) at 40 μmol m^-2^ s^-1^ PPFD. Bars labeled with different letters differ significantly between treatments (Tukey’s HSD test, *P* < 0.05).

Romaine lettuce showed distinct growth responses to the different light colors (**Supplementary Figures S2**–**S4**). In plants grown under downward lighting without upward lighting, the white LEDs yielded the highest total leaf fresh weights, and the blue LEDs produced the lowest total fresh weight (**Figure 4A**); this corresponded to the results for photosynthetic rate in the most newly expanded leaves of lettuce grown only with downward lighting (**Figure 3A**). Moreover, marketable leaf biomass, which represents the remaining leaves after removal of the outer senesced leaves, showed the same trend as total fresh weight (**Figure 4A**). Root mass generally varied little between treatments, although it was significantly greater with white LEDs than with red or blue LEDs in the absence of supplemental lighting. Other growth parameters, including leaf number, total leaf area, and root fresh and dry weights, were also greatest in plants grown under white LEDs (**Supplementary Table S3**). The wastage rate, which equaled the difference between the total and marketable leaf fresh weights, was higher in plants grown under white LEDs than in plants grown under red or blue LEDs without supplemental lighting (**Figure 5A**).

White or red upward lighting increased the total leaf fresh weight compared with plants grown without upward lighting, and the difference was significant with 16 days of supplemental lighting (**Figures 4B,C**). Moreover, the white and red supplemental lighting significantly increased marketable leaf fresh weight, leading to significantly lower wastage rates, especially in the 16-day treatments (**Figures 5B,C**). However, blue supplemental lighting made no significant difference (**Figures 4D** and **5D**).

### Ascorbic Acid and Nitrate Contents of the Romaine Lettuce

**FIGURE 6:**
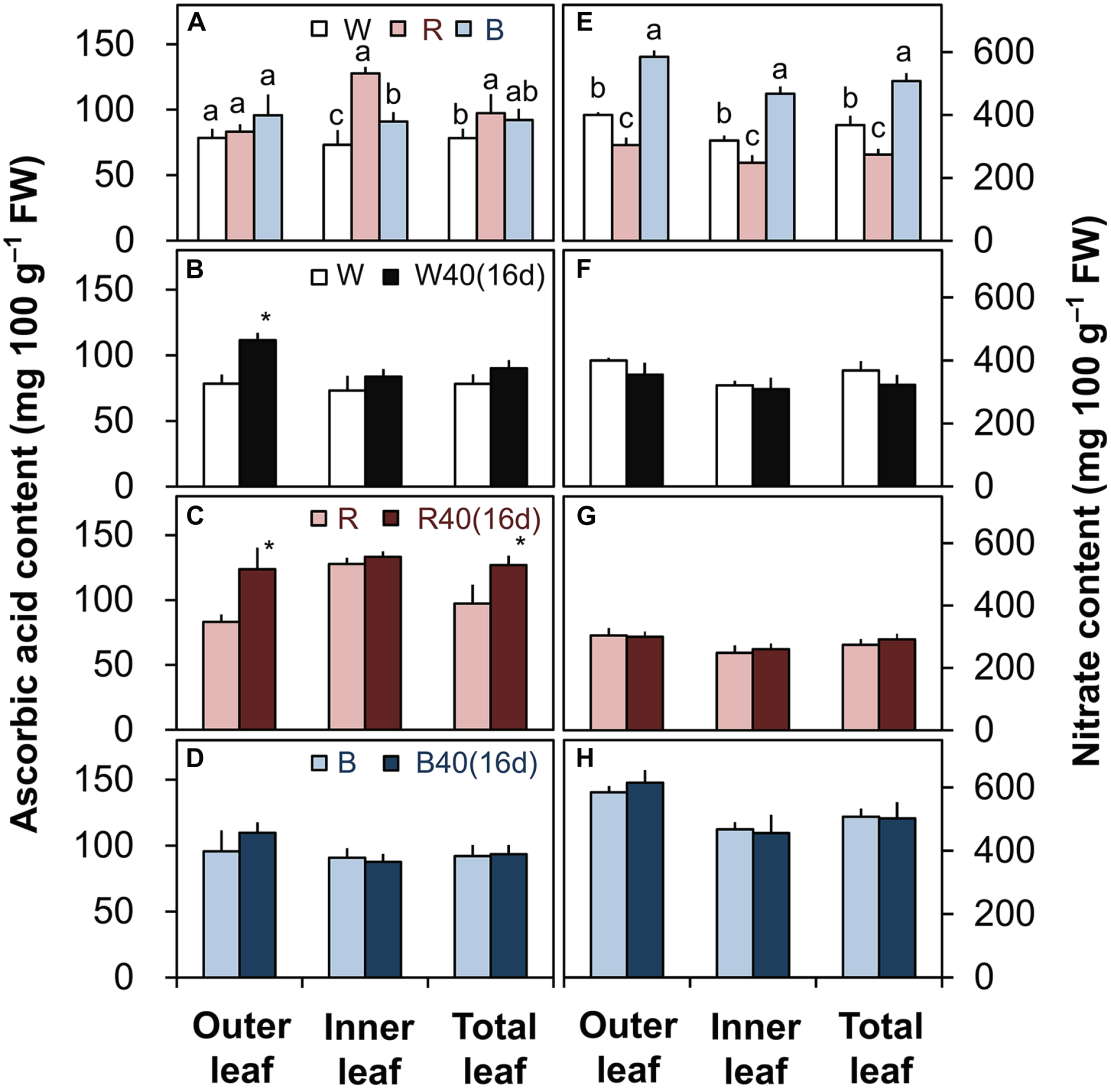
**Ascorbic acid **(A–D)** and nitrate contents **(E–H)** in the outer leaves, inner leaves, and total leaves of lettuce plants grown under downward lighting by white (W), red (R), or blue (B) LEDs: **(A,E)** without or **(B–D,F–H)** with supplemental upward lighting at 40 μmol m^-2^ s^-1^ PPFD for 16 days (“16d”).** Data represent means ± SD (*n* = 3 to 5). **(A,E)** Bars labeled with different letters differ significantly among the same plant parts (Tukey’s HSD test, *P* < 0.05). **(B–D,F–H)** Bars labeled with “^∗^” differ significantly between treatments (Student’s *t*-test, *P* < 0.05).

The ascorbic acid and nitrate contents were greatly influenced by the different light colors without upward lighting. The ascorbic acid content was generally highest under red LEDs, followed by blue and then white LEDs (**Figure 6A**). Conversely, the nitrate content of lettuce was significantly lower in leaves grown under red LEDs than in leaves grown under white or blue LEDs (**Figure 6E**).

Supplemental lighting significantly increased the ascorbic acid content in the outer leaves with white LEDs and in the outer and total leaves with red LEDs (**Figures 6B,C**), but did not significantly affect the nitrate content (**Figures 6F,G**). Supplemental lighting with blue LEDs did not significantly affect either (**Figures 6D,H**).

## Discussion

### White LEDs had a Greater Effect on Plant Biomass Production than Red or Blue LEDs

Romaine lettuces grown under white LEDs had significantly greater total and marketable leaf biomass than those grown under red or blue LEDs (**Figure 4A**), indicating that white light was a superior light source for the production of romaine lettuce in PFALs. Generally, there are two methods to generate white light: single-chip and multi-chip LEDs. Single-chip white LEDs include (1) a blue LED + a yellow phosphor (the most common type), (2) a blue LED + red and green phosphors, and (3) a near-ultraviolet LED + red, green, and blue phosphors. Multi-chip white LEDs include (1) red + green + blue LEDs and (2) blue + green + orange LEDs (Damilano et al., 2001; Taguchi, 2003). The white LEDs used in the present study combined a blue LED with a yellow phosphor (**Supplementary Figure S1A**). The results with the white LEDs in the present study suggest that combining light wavelengths could have a greater impact on plant production than the use of red and/or blue LEDs, which have been believed to be useful for plant cultivation in PFALs (e.g., Lee et al., 2010; Son and Oh, 2013). This hypothesis is supported by previous studies which showed that the dry mass of lettuce and spinach grown under only red light was significantly lower than that under white light, which provided both red and blue wavelengths (Yorio et al., 2001), and leaf fresh weight of lettuce grown under combined red and blue LEDs was significantly lower than under a combination of red, blue, and white LEDs (Lin et al., 2013). An increasing number of white LEDs are being manufactured for use in home lighting, resulting in reduced prices, and thus white LEDs might offer a good compromise between cost and optimal spectral characteristics as a light source for commercial plant productions in PFALs.

The different light colors influenced the ascorbic acid and nitrate contents in romaine lettuce (**Figures 6A,E**). Plants grown under red LEDs had the highest ascorbic acid content and the lowest nitrate content. If growers demand a higher ascorbic acid content or a lower nitrate content, red light could therefore help them meet their requirements. In PFALs, it is possible to match the light source to the production target. The levels of functional nutritional components such as ascorbic acid, alpha-carotene, and phenolic compounds can be increased by treatment with UV light (Xie et al., 2015) or red light (Bliznikas et al., 2012) during the late stages of cultivation. Therefore, in the future, it may be possible to achieve high plant yields with a high content of ascorbic acid or other nutrients by growing plants under white light and supplying red light during the late cultivation stage (e.g., 1 week before harvest) or directly under white light with supplemental red light.

### Supplemental Upward Lighting can Improve Plant Growth by Retarding Senescence of the Outer Leaves as Well as by Increasing Photosynthesis Rate

Plant cultivation at the high density used inside PFALs increases the annual production capacity per unit area (Kozai, 2013b). However, the outer leaves of plants grown at this high plant density cannot receive sufficient light from above and thus senesce faster. Our results confirm this hypothesis: the chlorophyll content and the maximum potential photochemical efficiency (*F*_v_/*F*_m_) both decreased drastically in the outer leaves without supplemental upward lighting (**Figures 2A,E**), which are typical phenomena when leaves senesce (Thimann and Satler, 1979; Wingler et al., 2004). However, our results clearly show that the upward lighting maintained a higher chlorophyll content (**Figures 2B,C**) and higher *F*_v_/*F*_m_ (**Figures 2F,G**) in the outer leaves, indicating retardation of senescence. Moreover, it was apparent that the supplemental lighting promoted photosynthesis in the outer leaves, whereas the plants without supplemental lighting had a negative carbon balance (**Figure 3B**). This interpretation is supported by the higher nitrogen content in the outer leaves of plants with supplemental lighting (**Supplementary Table S2**). Nitrogen is a major component of stromal enzymes and thylakoid proteins, so its availability strongly affects the plant’s photosynthetic capacity (Evans, 1989; Yamori et al., 2011). These results demonstrate that supplemental upward lighting both retarded leaf senescence and improved photosynthesis in the outer leaves, and that this increased plant yields (**Figures 4B,C**). Given the higher plant production, we propose that a novel cultivation system should be developed that includes supplemental upward lighting from below the plants to optimize the light conditions in PFALs. However, it will be necessary to perform additional research to determine whether the best results can be obtained with a single light color or a combination of colors.

It should also be noted that the effect of supplemental upward lighting could differ among plant species and light colors, as well as being a function of the plant qualities (e.g., total biomass, nutrient content) the breeder prioritizes. In the present study, the plants grown under blue LEDs grew more erect (**Supplementary Figure S4**, **Supplementary Table S4**) and therefore could not efficiently absorb the upward lighting (**Figures 2D,H and 4D**). Thus, it is necessary to select suitable plant species and light sources for this cultivation method. Further research will be needed to optimize the cultivation system using supplemental upward lighting.

### Economic Benefit Analysis of Supplemental Upward Lighting

Because of the vertical cultivation pattern (**Figure 1**), a PFAL with 10 tiers of plants can have an annual production capacity of leafy vegetables that is 90–117 times the values that can be achieved in an open field (Kozai, 2013b). The largest PFAL in Japan (Spread Inc., Kyoto, Japan) can produce 21,000 lettuce heads per day, and the largest one in Taiwan (Yasai Corp., Taiwan) can produce 40,000 per day. Although these production rates are extremely high, yield losses caused by outer leaf senescence are also large. Based on total and marketable leaf fresh weights of 153.7 and 134.0 g, respectively, under white downward lighting from above with no supplemental upward lighting (**Supplementary Table S3**), senescence of the outer leaves in these operations could cause losses of 413.7 kg FW/day in the Japanese PFAL and 788.0 kg FW/day in the Taiwanese PFAL. The present results clearly show that the wastage rate can be significantly decreased by the use of supplemental upward lighting (**Figures 5B,C**). Thus, supplemental upward lighting to delay senescence of outer leaves could be an attractive way to solve this problem while also decreasing labor requirements to remove the senesced leaves.

To analyze the economic benefits of the supplemental upward lighting, it is necessary to account for the energy cost of the lighting. **Supplementary Table S5** provides this comparison based on an electricity cost of 17.49 JPY/kW h, and the difference between the net retail price of the lettuce plants with and without supplemental upward lighting (i.e., the net income of the supplemental upward lighting). In the 9-day treatments, white LEDs (11.5 JPY/plant) produced a higher net income than red LEDs (1.62 JPY/plant), and blue LEDs (–2.27 JPY/plant). The results were similar for the 16-day treatment: the highest net income was again obtained with white LEDs (40.3 JPY/plant) followed by red LEDs (20.9 JPY/plant) and blue LEDs (-3.56 JPY/plant). White LEDs with 16 days of supplemental upward white lighting yielded the highest biomass (158.0 g/plant) and the highest net income (40.3 JPY/plant). The net incomes calculated in this analysis don’t include the savings that result from decreasing the labor cost for trimming of senesced leaves during packing, and thus the net benefit would be greater than our estimates. Although the 9-day supplemental treatments would reduce the electricity costs, plant biomass and net income increased less than in the 16-day treatments (**Supplementary Table S5**). Thus, in order to increase production and net income, it is better to provide supplemental upward lighting for 16 days or even longer (e.g., from transplanting to harvest). However, determining the optimal duration and spectral characteristics of the supplemental light remains a challenge for future research.

## Author Contributions

GZ and WY conceived and designed the experiments. GZ performed the experiments. GZ and WY prepared the manuscript, and WY, GZ, SS, TK, and MT contributed extensively to its finalization.

## Conflict of Interest Statement

The authors declare that the research was conducted in the absence of any commercial or financial relationships that could be construed as a potential conflict of interest.
