# Supplementary material for: Supplemental Upward Lighting from Underneath to Obtain Higher Marketable Lettuce (Lactuca sativa) Leaf Fresh Weight by Retarding Senescence of Outer Leaves
Source: Front Plant Sci. 2015 Dec 14;6:1110. doi: 10.3389/fpls.2015.01110 (PMC4677251; doi:10.3389/fpls.2015.01110)
Supplement: Supplementary file 1 [file Data_Sheet_1.PDF]

Supplementary Material:

Supplemental upward lighting from underneath to obtain higher marketable lettuce (*Lactuca sativa*) leaf fresh weight by retarding senescence of outer leaves

Geng Zhang, Shanqi Shen, Michiko Takagaki, Toyoki Kozai, Wataru Yamori*

***Correspondence:**

Wataru Yamori:

wataru.yamori@chiba-u.jp

1 SUPPLEMENTARY DATA

Supplementary Tables S1-5

Supplementary Figures S1-4

2 SUPPLEMENTARY TABLES AND FIGURES

Supplementary Table S1. Total chlorophyll content and maximum quantum yield (F_v/F_m) in lettuce leaves from the six layers of leaves in plants grown under illumination from above by white (W), red (R), or blue (B) LEDs, without or with supplemental upward lighting for 9 days or 16 days at 40 µmol m^{-2} s^{-1} PPFD. Data represent means ± SD (n = 3 to 5). Data in the first three rows expressed the plants grown with illumination from above by white, red, or blue LEDs without any supplemental upward lighting, means followed by different letters differ significantly (Tukey's HSD test, $P < 0.05$) among the three light colors. For the following rows, different letters follow the values of the rows within the same light color refer to a significant difference between two PPFDs and three treatment durations (Tukey's HSD test, $P < 0.05$).

Light source (LEDs)	PPFD (µmol m^{-2} s^{-1})	Supplemental upward lighting treatment (day)	Total chlorophyll content (g m^{-2})					
			1st layer	2nd layer	3rd layer	4th layer	5th layer	6th layer
White	200-0	0	0.039 ± 0.029 b	0.093 ± 0.023 c	0.155 ± 0.032 b	0.230 ± 0.016 b	0.294 ± 0.015 a	0.301 ± 0.007 a
Red	200-0	0	0.095 ± 0.024 a	0.148 ± 0.022 b	0.191 ± 0.019 b	0.273 ± 0.019 a	0.292 ± 0.016 a	0.298 ± 0.012 a
Blue	200-0	0	0.121 ± 0.031 a	0.188 ± 0.025 a	0.236 ± 0.034 a	0.256 ± 0.032 ab	0.253 ± 0.015 b	0.265 ± 0.027 b
White	200-0	0	0.039 ± 0.029 b	0.093 ± 0.023 b	0.155 ± 0.032 b	0.230 ± 0.016 b	0.294 ± 0.015 a	0.301 ± 0.007 a
	200-40	9	0.091 ± 0.010 a	0.158 ± 0.028 a	0.204 ± 0.024 a	0.265 ± 0.020 a	0.299 ± 0.009 a	0.304 ± 0.007 a
	200-40	16	0.101 ± 0.018 a	0.186 ± 0.027 a	0.223 ± 0.013 a	0.271 ± 0.032 a	0.301 ± 0.013 a	0.303 ± 0.017 a
Red	200-0	0	0.095 ± 0.024 b	0.148 ± 0.022 b	0.191 ± 0.019 b	0.273 ± 0.019 a	0.292 ± 0.016 a	0.298 ± 0.012 a
	200-40	9	0.147 ± 0.022 a	0.181 ± 0.017 a	0.225 ± 0.017 a	0.285 ± 0.012 a	0.292 ± 0.014 a	0.299 ± 0.006 a
	200-40	16	0.168 ± 0.019 a	0.206 ± 0.017 a	0.250 ± 0.010 a	0.291 ± 0.013 a	0.302 ± 0.016 a	0.308 ± 0.020 a
Blue	200-0	0	0.121 ± 0.031 a	0.188 ± 0.025 a	0.236 ± 0.034 a	0.256 ± 0.032 a	0.253 ± 0.015 a	0.265 ± 0.027 a
	200-40	9	0.131 ± 0.018 a	0.193 ± 0.026 a	0.235 ± 0.017 a	0.251 ± 0.024 a	0.264 ± 0.030 a	0.265 ± 0.026 a
	200-40	16	0.132 ± 0.013 a	0.189 ± 0.024 a	0.237 ± 0.016 a	0.256 ± 0.018 a	0.266 ± 0.024 a	0.261 ± 0.018 a

Light source (LEDs)	PPFD (μmol m^{-2} s^{-1})	Supplemental upward lighting treatment (day)	F_v/F_m					
			1st layer	2nd layer	3rd layer	4th layer	5th layer	6th layer
White	200-0	0	0.042 ± 0.025 ab	0.288 ± 0.101 a	0.438 ± 0.150 a	0.659 ± 0.094 a	0.714 ± 0.074 a	0.721 ± 0.063 a
Red	200-0	0	0.173 ± 0.077 a	0.270 ± 0.032 a	0.390 ± 0.141 a	0.683 ± 0.038 a	0.663 ± 0.038 a	0.705 ± 0.035 a
Blue	200-0	0	0.172 ± 0.076 a	0.328 ± 0.122 a	0.513 ± 0.179 a	0.628 ± 0.033 a	0.715 ± 0.031 a	0.715 ± 0.083 a
White	200-0	0	0.042 ± 0.025 c	0.288 ± 0.101 b	0.438 ± 0.150 b	0.659 ± 0.094 a	0.714 ± 0.074 a	0.721 ± 0.063 a
	200-40	9	0.204 ± 0.079 b	0.539 ± 0.040 a	0.537 ± 0.099 ab	0.685 ± 0.079 a	0.748 ± 0.048 a	0.731 ± 0.056 a
	200-40	16	0.519 ± 0.071 a	0.636 ± 0.066 a	0.664 ± 0.044 a	0.738 ± 0.063 a	0.748 ± 0.060 a	0.761 ± 0.033 a
Red	200-0	0	0.173 ± 0.077 b	0.270 ± 0.032 b	0.390 ± 0.141 b	0.683 ± 0.038 a	0.663 ± 0.038 a	0.705 ± 0.035 a
	200-40	9	0.312 ± 0.094 b	0.585 ± 0 .090 a	0.623 ± 0.126 a	0.690 ± 0.029 a	0.685 ± 0.052 a	0.717 ± 0.031 a
	200-40	16	0.592 ± 0.078 a	0.648 ± 0.046 a	0.629 ± 0.049 a	0.699 ± 0.033 a	0.715 ± 0.050 a	0.736 ± 0.020 a
Blue	200-0	0	0.172 ± 0.076 a	0.328 ± 0.122 a	0.513 ± 0.179 a	0.628 ± 0.033 a	0.715 ± 0.031 a	0.715 ± 0.083 a
	200-40	9	0.143 ± 0.025 a	0.380 ± 0.115 a	0.553 ± 0.128 a	0.561 ± 0.092 a	0.701 ± 0.084 a	0.737 ± 0.038 a
	200-40	16	0.237 ± 0.035 a	0.330 ± 0.174 a	0.559 ± 0.106 a	0.624 ± 0.086 a	0.718 ± 0.051 a	0.745 ± 0.042 a

Supplementary Table S2. Effects of light color and duration of supplemental upward lighting on outer (3rd layer) and inner (6th layer) leaves properties (LMA, leaf mass per area; nitrogen content; C/N, the carbon to nitrogen ratio) in romaine lettuce. Data represent means ± SD (*n* = 5). Data in the first three rows expressed the plants grown with illumination from above by white, red, or blue LEDs without any supplemental upward lighting, means followed by different letters differ significantly (Tukey's HSD test, $P < 0.05$) among three light colors. For the following rows, different letters follow the values of the rows within same light color refer to a significant difference between two PPFDs (Student's *t*-test, $P < 0.05$).

Light source (LEDs)	PPFD (μmol m^{-2} s^{-1})	Supplemental upward lighting treatment (day)	Outer leaf			Inner leaf		
			LMA (g m^{-2})	Nitrogen (g m^{-2})	C/N (g g^{-1})	LMA (g m^{-2})	Nitrogen (g m^{-2})	C/N (g g^{-1})
White	200-0	0	13.2 ± 1.5 a	1.24 ± 0.27 b	10.05 ± 1.65 a	14.3 ± 4.2 ab	2.09 ± 0.57 a	6.09 ± 1.09 a
Red	200-0	0	12.3 ± 0.8 a	1.80 ± 0.36 a	6.79 ± 1.45 b	13.3 ± 1.1 b	2.13 ± 0.30 a	6.38 ± 0.96 a
Blue	200-0	0	12.8 ± 1.4 a	1.97 ± 0.31 a	6.12 ± 0.67 b	15.3 ± 0.8 a	2.48 ± 0.26 a	5.90 ± 0.34 a
White	200-0	0	13.2 ± 1.5 a	1.24 ± 0.27 b	10.05 ± 1.65 a	14.3 ± 4.2 a	2.09 ± 0.57 a	6.09 ± 1.09 a
	200-40	16	13.9 ± 1.8 a	1.58 ± 0.12 a	7.90 ± 1.17 a	16.0 ± 3.1 a	2.56 ± 0.58 a	6.56 ± 1.09 a
Red	200-0	0	12.3 ± 0.8 a	1.80 ± 0.36 a	6.79 ± 1.45 a	13.3 ± 1.1 a	2.13 ± 0.30 a	6.38 ± 0.96 a
	200-40	16	12.6 ± 1.9 a	2.04 ± 0.59 a	6.05 ± 1.10 a	14.0 ± 1.5 a	1.92 ± 0.26 a	6.99 ± 0.96 a
Blue	200-0	0	12.8 ± 1.4 a	1.97 ± 0.31 a	6.12 ± 0.67 a	15.3 ± 0.8 a	2.48 ± 0.26 a	5.90 ± 0.34 a
	200-40	16	12.3 ± 1.2 a	2.10 ± 0.27 a	5.77 ± 0.65 a	12.6 ± 2.9 a	2.00 ± 0.28 b	5.92 ± 0.61 a

Supplementary Table S3. Effects of light color and duration of supplemental upward lighting on shoot angle, leaf number, total leaf area, root length, leaf and root fresh weights (FW), leaf and root dry weights (DW), and leaf/root (DW) at harvest time. Data represent means ± SD (*n* = 3 to 5). Data in the first three rows expressed the plants grown with illumination from above by white, red, or blue LEDs without any supplemental upward lighting, means followed by different letters differ significantly (Tukey's HSD test, P < 0.05) among three light colors. For the following rows, different letters follow the values of the rows within the same light color refer to a significant difference between two PPFDs and three treatment durations (Tukey's HSD test, P < 0.05).

Light source (LEDs)	PPFD (μmol m^{-2} s^{-1})	Supplemental upward lighting treatment (day)	Shoot Angle (º)	Leaf number	Total leaf area (m^2)	Root length (cm)	Fresh weight (g)			Dry weight (g)		Leaf/Root (DW)
							Total leaf	Marketable leaf	Root	Total leaf	Root	
White	200-0	0	55.4 ± 0.5 b	32.2 ± 1.3 a	0.302 ± 0.022 a	28.5 ± 4.4 b	153.7 ± 9.9 a	134.0 ± 8.2 a	11.0 ± 0.6 a	4.05 ± 0.12 a	0.626 ± 0.065 a	6.52 ± 0.70 a
Red	200-0	0	43.5 ± 3.2 c	31.6 ± 1.1 a	0.234 ± 0.013 b	32.0 ± 2.4 b	119.8 ± 6.3 b	110.0 ± 6.2 b	9.0 ± 0.9 b	3.55 ± 0.40 ab	0.520 ± 0.091 ab	6.92 ± 0.71 a
Blue	200-0	0	70.6 ± 1.3 a	21.4 ± 1.3 b	0.164 ± 0.009 c	42.7 ± 6.9 a	77.1 ± 1.0 c	71.5 ± 1.3 c	8.2 ± 0.7 b	3.35 ± 0.24 b	0.430 ± 0.084 b	8.00 ± 1.47 a
White	200-0	0	55.4 ± 0.5 a	32.2 ± 1.3 a	0.302 ± 0.022 b	28.5 ± 4.4 a	153.7 ± 9.9 b	134.0 ± 8.2 b	11.0 ± 0.6 a	4.05 ± 0.12 b	0.626 ± 0.065 a	6.52 ± 0.70 a
	200-40	9	——	33.0 ± 1.2 a	——	——	159.3 ± 6.7 b	141.9 ± 6.7 b	11.2 ± 0.6 a	4.14 ± 0.17 ab	0.658 ± 0.049 a	6.31 ± 0.43 a
	200-40	16	46.0 ± 2.3 b	33.6 ± 1.8 a	0.390 ± 0.023 a	32.6 ± 4.1 a	177.4 ± 12.3 a	158.0 ± 11.4 a	11.2 ± 1.3 a	4.42 ± 0.36 a	0.644 ± 0.070 a	6.90 ± 0.67 a
Red	200-0	0	43.5 ± 3.2 a	31.6 ± 1.1 a	0.234 ± 0.013 b	32.0 ± 2.4 a	119.8 ± 6.3 b	110.0 ± 6.2 b	9.0 ± 0.9 a	3.55 ± 0.40 a	0.520 ± 0.091 a	6.92 ± 0.71 a
	200-40	9	——	31.6 ± 1.5 a	——	——	124.9 ± 7.2 b	117.4 ± 7.2 b	8.8 ± 0.9 a	3.81 ± 0.40 a	0.516 ± 0.058 a	7.39 ± 0.44 a
	200-40	16	44.0 ± 3.4 a	32.2 ± 1.6 a	0.309 ± 0.002 a	31.0 ± 2.9 a	138.1 ± 7.7 a	132.1 ± 8.4 a	9.3 ± 1.3 a	4.11 ± 0.32 a	0.542 ± 0.067 a	7.63 ± 0.45 a
Blue	200-0	0	70.6 ± 1.3 a	21.4 ± 1.3 a	0.164 ± 0.009 a	42.7 ± 6.9 a	77.1 ± 1.0 a	71.5 ± 1.3 a	8.2 ± 0.7 a	3.35 ± 0.24 a	0.430 ± 0.084 a	8.00 ± 1.47 a
	200-40	9	——	20.8 ± 1.3 a	——	——	78.4 ± 1.0 a	71.2 ± 1.4 a	8.3 ± 1.4 a	3.32 ± 0.19 a	0.448 ± 0.070 a	7.54 ± 1.17 a
	200-40	16	70.0 ± 3.5 a	21.0 ± 1.6 a	0.171 ± 0.007 a	42.0 ± 7.3 a	79.2 ± 2.6 a	71.2 ± 2.5 a	7.9 ± 0.9 a	3.32 ± 0.23 a	0.454 ± 0.072 a	7.42 ± 0.99 a

Supplementary Table S4. Light intensity which was received by the outer leaves of plants grown with illumination from above by white, red, or blue LEDs with supplemental upward lighting. The LEDs were located at the panels on the growth beds. The photosynthetic photon flux density (PPFD) was measured by R-2D color acetate film (R-2D, Taisei Chemical Industries, Tokyo, Japan) at the height of the outer leaves of lettuce grown with illumination from above by white, red, or blue LEDs with supplemental upward lighting. After 14 days of being illuminated by supplemental upward lighting, the R-2D color acetate films were collected for calculation of the light intensity by the equation: [(833.3−416.7 × (log10D/Do × 100)/(14 × 24 × 60 × 60)] × 106, Do = 1.982 (value of film before exposing to the light), D represents the value of film after being exposed to the white, red, or blue supplemental upward lighting. Means followed by different letters differ significantly (Tukey's HSD test, $P < 0.05$) among three light colors.

Supplemental upward lighting source (LED tapes)	PPFD at 4.0 cm distance from LEDs (μmol m^{-2} s^{-1})	Shoot Angle (º)	PPFD at the height of the outer leaves (μmol m^{-2} s^{-1})
White	40	55.4 ± 0.5 b	37.2 ± 1.7 a
Red	30	43.5 ± 3.2 c	23.2 ± 1.7 b
Blue	40	70.6 ± 1.3 a	6.42 ± 1.1 c

Supplementary Table S5. Evaluation of the feasibility of supplemental upward lighting with different durations. For each treatment the plant density was 37 plants m^{-2} and the light period was 16 hour. The power of different LED tapes was: white LEDs tape (20.6 W), blue LEDs tape (25.1 W), and red LEDs tape (62.8 W). Based on local surveys, the retail price of lettuce was 200 JPY/100 g and the electricity bill was 17.49 JPY/KW h. (A) Retail price per plant was calculated as: A = marketable leaf fresh weight/100 × 200; (B) the electricity bill for supplemental upward lighting per plant was calculated as: B = electricity consumption of LED × 16/1000/12 × treatment days × 17.49, where 16 was the photoperiod and 12 was the number of plants illuminated by supplemental LEDs from underneath the plant; net retail price per plant was calculated as A minus B, and net income of the supplemental upward lighting per plant was obtained by the difference between the net retail price of the lettuce plants with and without supplemental lighting. Different letters follow the values of the rows within same light color refer to a significant difference between two PPFDs and three treatment durations (Tukey's HSD test, $P < 0.05$).

Light source (LEDs)	PPFD (μmol m^{-2} s^{-1})	Supplemental upward lighting treatment (day)	Marketable leaf fresh weight (g)	(A) Retail price (JPY/plant)	(B) Electricity bill of supplemental upward lighting (JPY/plant)	Net retail price (JPY/plant)	Net income of the supplemental upward lighting (JPY/plant)
White	200-0	0	134.0 ± 8.2 b	268.0 ± 16.4 c	0.00 ± 0.00 c	268.0 ± 0.0 c	0.00 ± 0.00 c
	200-40	9	141.9 ± 6.7 b	283.8 ± 13.4 b	4.32 ± 0.03 b	279.5 ± 0.0 b	11.5 ± 0.0 b
	200-40	16	158.0 ± 11.4 a	315.9 ± 22.7 a	7.69 ± 0.06 a	308.2 ± 0.1 a	40.3 ± 0.10 a
Red	200-0	0	110.0 ± 6.2 b	219.9 ± 12.4 c	0.00 ± 0.00 c	219.9 ± 0.0 c	0.00 ± 0.00 c
	200-40	9	117.4 ± 7.2 b	234.7 ± 14.4 b	13.2 ± 0.0 b	221.6 ± 0.0 b	1.62 ± 0.03 b
	200-40	16	132.1 ± 8.4 a	264.2 ± 16.9 a	23.4 ± 0.10 a	240.8 ± 0.1 a	20.9 ± 0.1 a
Blue	200-0	0	69.7 ± 1.3 a	139.4 ± 2.7 a	0.00 ± 0.00 c	139.4 ± 0.0 a	0.00 ± 0.00 a
	200-40	9	71.2 ± 1.4 a	142.3 ± 2.7 b	5.27 ± 0.03 b	137.1 ± 0.0 b	-2.27 ± 0.03 b
	200-40	16	72.6 ± 2.5 a	145.2 ± 5.0 b	9.36 ± 0.05 a	135.8 ± 0.0 c	-3.56 ± 0.05 c

.

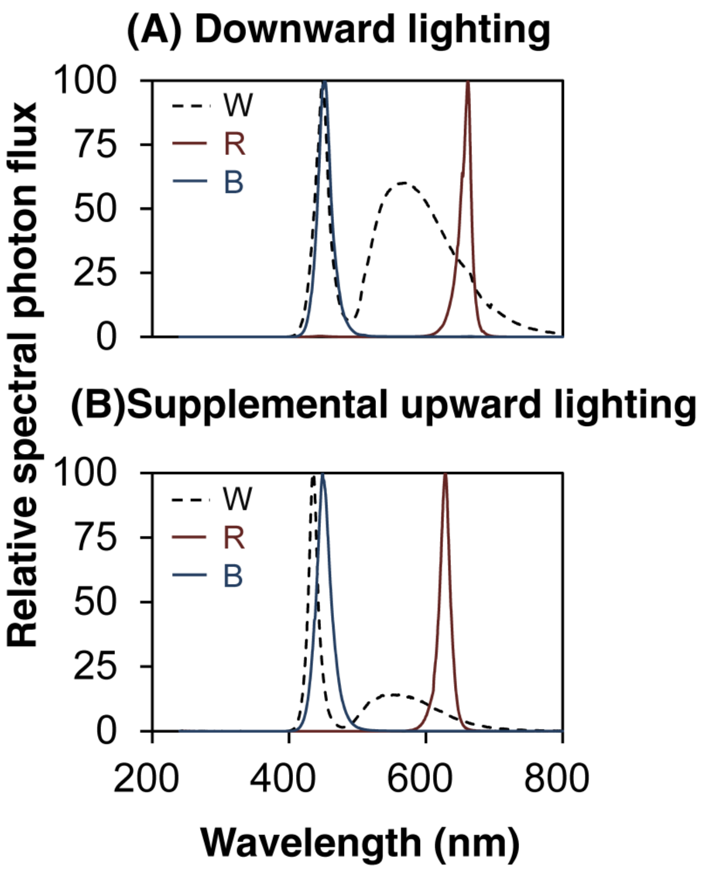

Supplementary Figure S1. The relative spectral photon flux of (A) downward lighting (top LEDs) and (B) supplemental upward lighting (bottom LEDs). The wavelengths of light sources were recorded at 240-800 nm with a spectrometer (SR9910-v7, irradiant Ltd., Tranent, UK). W, R, or B denotes white, red, or blue LEDs.

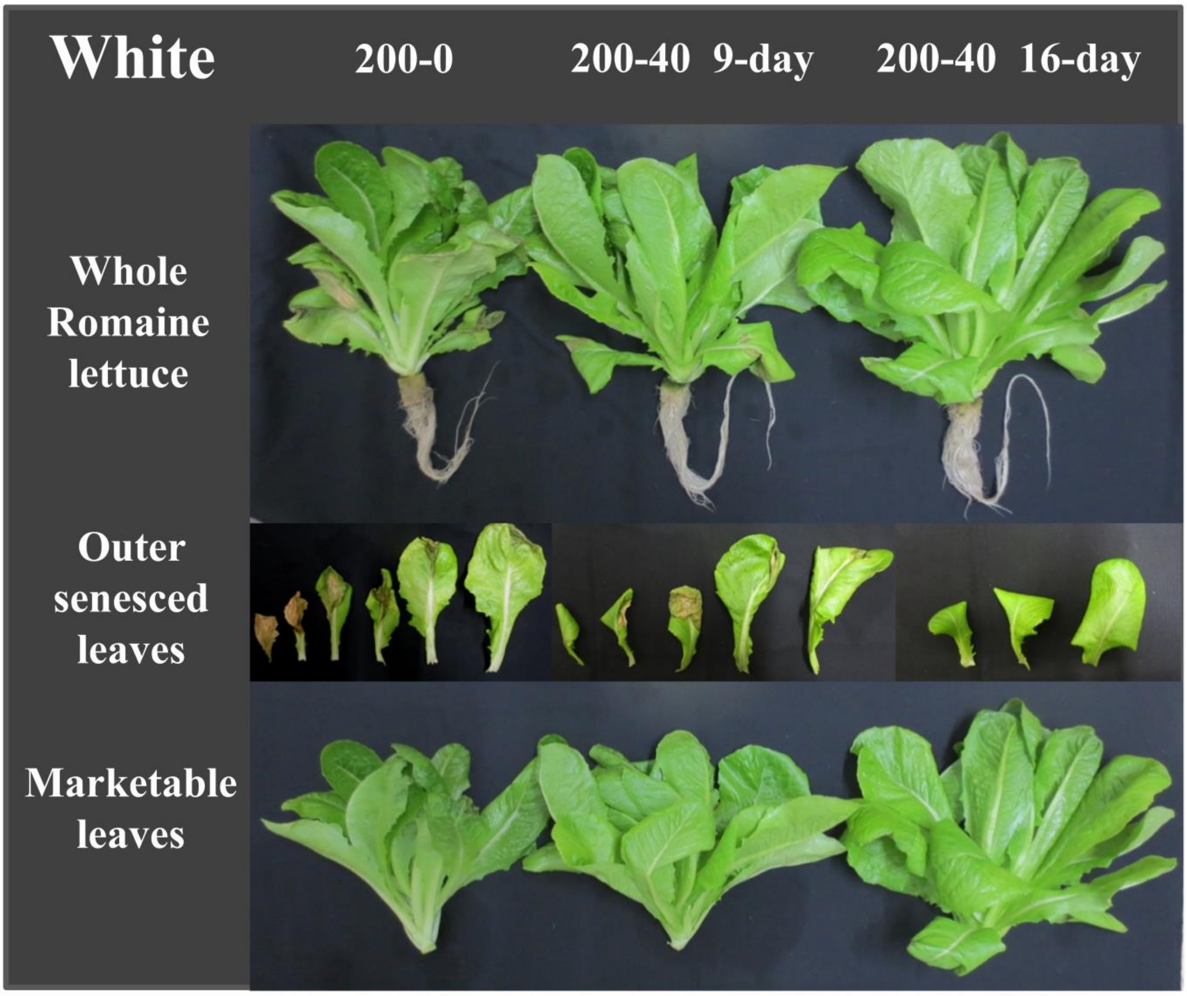

Supplementary Figure S2. Pictures of romaine lettuce under white light treatments at the harvest time. From left to right: 200 µmol m^{-2} s^{-1} downward lighting with 0 supplemental upward lighting; 200 µmol m^{-2} s^{-1} downward lighting with a 9-day treatment of 40 µmol m^{-2} s^{-1} supplemental upward lighting; 200 µmol m^{-2} s^{-1} downward lighting with a 16-day treatment of 40 µmol m^{-2} s^{-1} supplemental upward lighting.

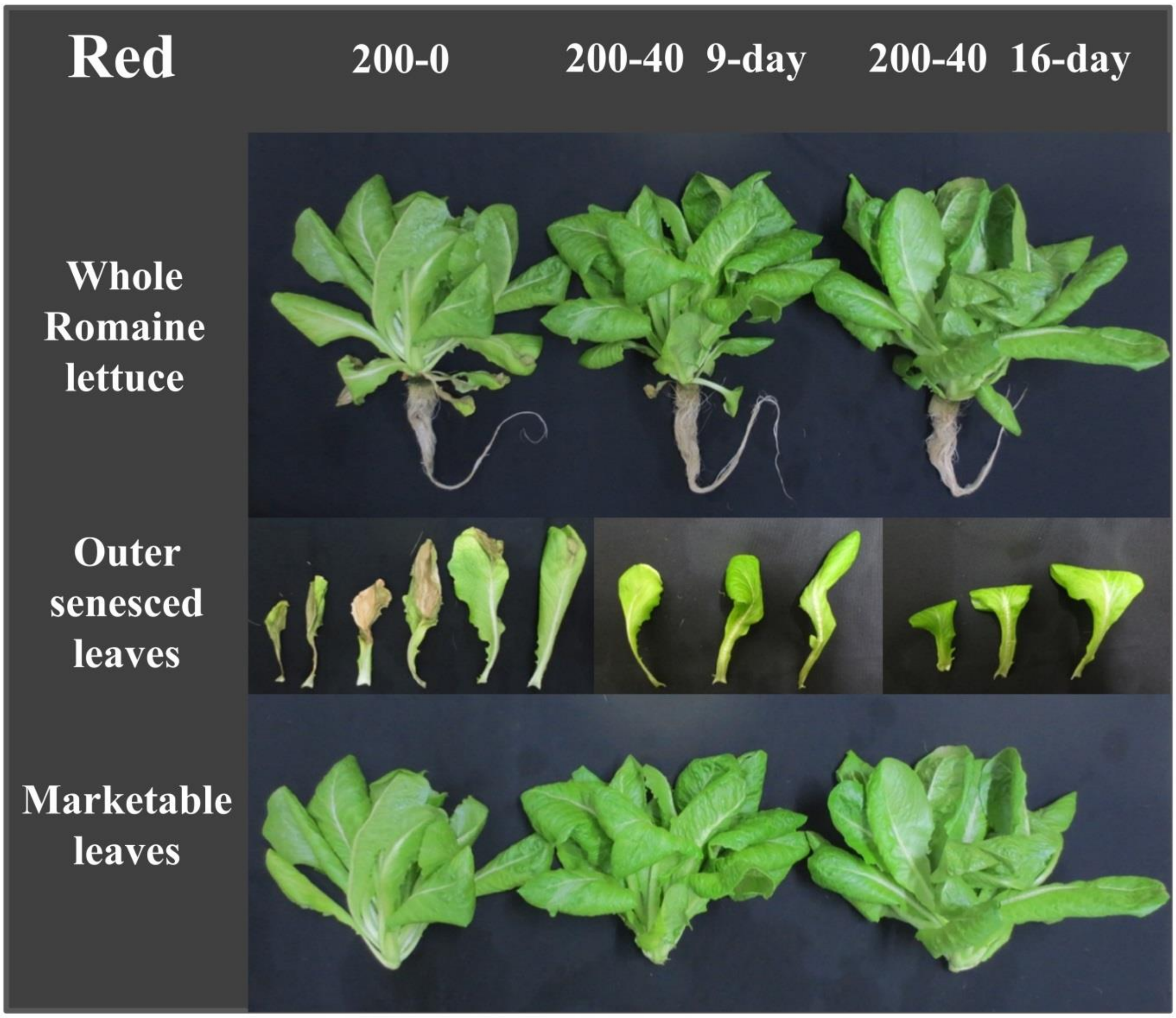

Supplementary Figure S3. Pictures of romaine lettuce under red light treatments at the harvest time. From left to right: 200 μmol m^{-2} s^{-1} downward lighting with 0 supplemental upward lighting; 200 μmol m^{-2} s^{-1} downward lighting with a 9-day treatment of 40 μmol m^{-2} s^{-1} supplemental upward lighting; 200 μmol m^{-2} s^{-1} downward lighting with a 16-day treatment of 40 μmol m^{-2} s^{-1} supplemental upward lighting.

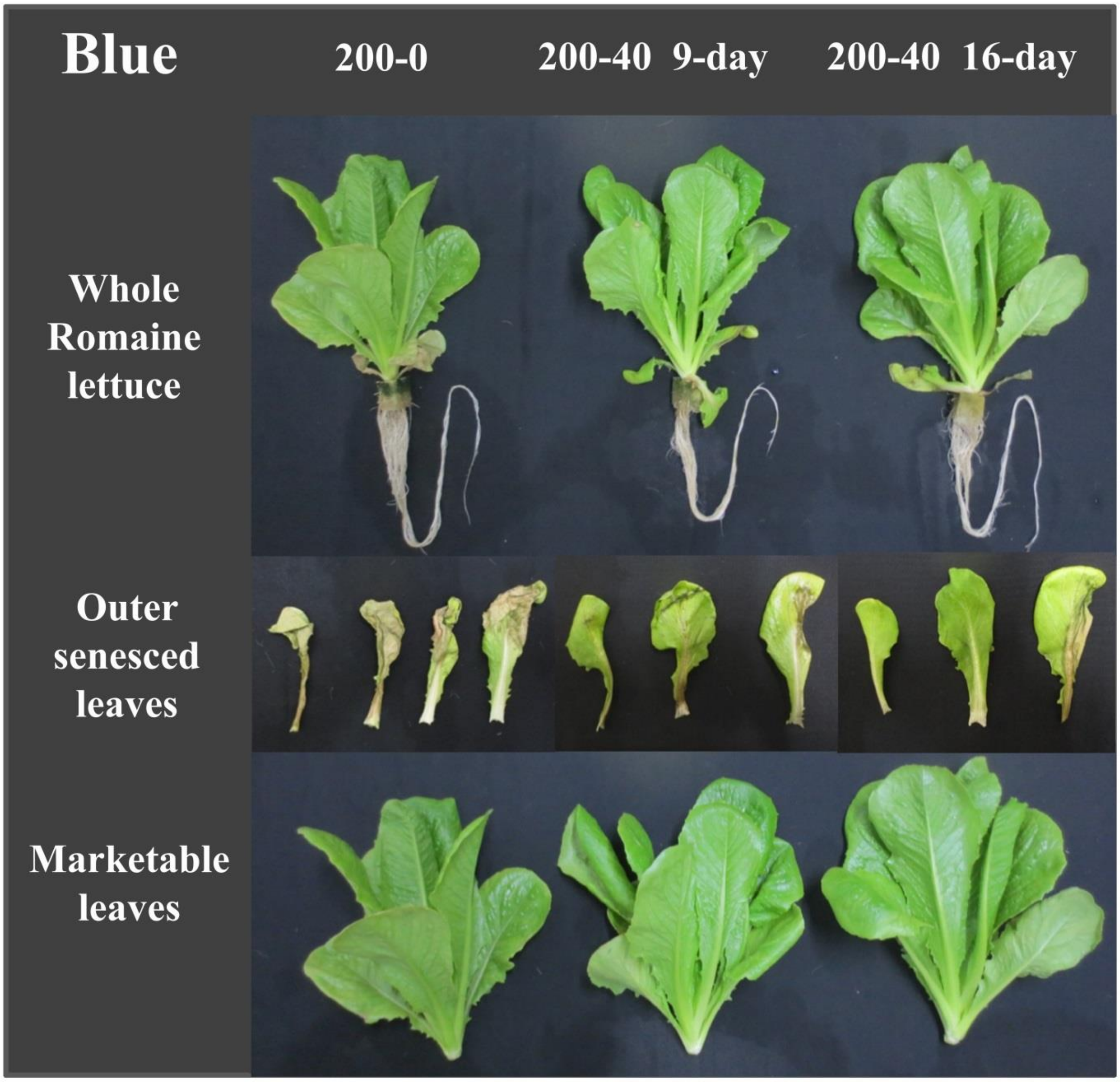

Supplementary Figure S4. Pictures of romaine lettuce under blue light treatments at the harvest time. From left to right: 200 µmol m^{-2} s^{-1} downward lighting with 0 supplemental upward lighting; 200 µmol m^{-2} s^{-1} downward lighting with a 9-day treatment of 40 µmol m^{-2} s^{-1} supplemental upward lighting; 200 µmol m^{-2} s^{-1} downward lighting with a 16-day treatment of 40 µmol m^{-2} s^{-1} supplemental upward lighting.
